# RF eigenfingerprints, an Efficient RF Fingerprinting Method in IoT Context

**DOI:** 10.3390/s22114291

**Published:** 2022-06-05

**Authors:** Louis Morge-Rollet, Frédéric Le Roy, Denis Le Jeune, Charles Canaff, Roland Gautier

**Affiliations:** 1 ENSTA Bretagne, Lab-STICC, CNRS, UMR 6285, F-29200 Brest, France; frederic.le_roy@ensta-bretagne.fr (F.L.R.); denis.le_jeune@ensta-bretagne.fr (D.L.J.); charles.canaff@ensta-bretagne.fr (C.C.); 2 Lab-STICC, Université de Bretagne Occidentale, CEDEX 3, F-29238 Brest, France; roland.gautier@univ-brest.fr

**Keywords:** RF fingerprinting, IoT networks security, eigenfaces, FPGA implementation

## Abstract

In IoT networks, authentication of nodes is primordial and RF fingerprinting is one of the candidates as a non-cryptographic method. RF fingerprinting is a physical-layer security method consisting of authenticated wireless devices using their components’ impairments. In this paper, we propose the RF eigenfingerprints method, inspired by face recognition works called eigenfaces. Our method automatically learns important features using singular value decomposition (SVD), selects important ones using Ljung–Box test, and performs authentication based on a statistical model. We also propose simulation, real-world experiment, and FPGA implementation to highlight the performance of the method. Particularly, we propose a novel RF fingerprinting impairments model for simulation. The end of the paper is dedicated to a discussion about good properties of RF fingerprinting in IoT context, giving our method as an example. Indeed, RF eigenfingerprint has interesting properties such as good scalability, low complexity, and high explainability, making it a good candidate for implementation in IoT context.

## 1. Introduction

Internet of Things (IoT) is one of the most important 21st century technologies. It connects smart devices such as sensors and actuators to the Internet. IoT is present everywhere from wearables (smart watches, etc.) to smart cities, impacting many domains such as transportation, energy, and agriculture, among others.

According to [1,2], security and privacy are two of the most important challenges in IoT networks. Nowadays, Internet security depends on cryptography ensuring confidentiality (AES, …), authenticity (RSA, …), integrity (SHA-256, …), and non-repudiation. However, Sankhe et al. [3] explained that classic cryptography algorithms such as RSA cannot be handled by IoT smart devices. Indeed, these devices are generally low-power technologies with small computation capacities. Thus, RF fingerprinting is one of the candidates to authenticate smart devices in IoT networks as non-cryptographic technology [4]. It consists of authenticating a device using their components’ impairments (carrier offset, I/Q imbalance, etc.). One of the interesting properties of RF fingerprinting is that signatures are considered as non-fungible. Historically, RF fingerprinting methods used expert hand-crafted features but are now considered as dependent on a priori assumptions [5]. Thus, recently, feature learning based on deep learning models has been widely developed for RF fingerprinting [3,5,6]. However, the main problem of using deep learning for RF fingerprinting is that it requires a lot of data for learning and has high computation complexity. New solutions must be developed to overcome these issues.

In this paper, we propose a novel method for RF fingerprinting called RF eigenfingerprints. Our proposal is a feature-learning RF fingerprinting method based on face recognition works, developed in the early 1990s, called eigenfaces. Our method allows to automatically learn features for RF fingerprinting, has low computation complexity, and requires few data for learning. The principal contributions of this paper are the following:  

1. We adapt the eigenfaces principles to RF fingerprinting domain. Furthermore, we develop a strong theoretical background of our approach.
2. We propose a novel baseband model for emitter impairments simulation, taking into account I/Q offset, I/Q imbalance, and power amplifier nonlinearity.
3. We develop a lightweight FPGA implementation of our features projection step on Digilent Zedboard (Xilinx Zynq-7000).
4. We present a methodology to interpret the features learned by our algorithm.  

Section 2 is dedicated to the state of the art presenting eigenfaces and RF fingerprinting. Section 3 presents the methodology for RF eigenfingerprints, and Section 4 presents different experiments. In Section 5, we discuss important properties for RF fingerprinting in IoT context, taking our method as example. Finally, Section 6 concludes this article.

## 2. State of the Art

### 2.1. Eigenfaces

**Figure 1:**
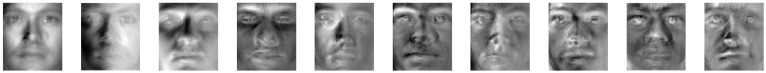
Eigenfaces example.

In 1987, M. Kirby and L. Sirovich [7,8] proposed the use of Karhunen–Loève transform to automatically learn important features for face recognition. The Karhunen–Loève transform, also called principal component analysis (PCA) in machine learning [9,10], is a transform that learns automatically on an orthogonal basis widely used for dimensionality reduction. In 1991, M. Turk and A. Pentland [11,12] developed a face recognition method called eigenfaces based on previously introduced works of Kirby and Sirovich. The selected eigenvectors learned by Karhunen–Loève transform are called eigenfaces. An example of eigenfaces learned on Yale Face dataset [13,14] is provided in Figure 1. (Figure 1 (The eigenfaces are learned using the extended Yale Face database B [13,14])). In addition to automatically learned features for face recognition, their method allows classification of known faces, rejection of unknown faces, and even detects that an image under investigation is not a face image. Their work is considered to be the first successful example of facial recognition technology. Furthermore, eigenfaces have influenced many face recognition works, such as fisherfaces [13], but also different domains under the name of eigenimages.

### 2.2. RF Fingerprinting

 As already explained, RF fingerprinting methods are part of non-cryptographic authentication [4] aiming to authenticate a device without using cryptography. On one hand, software-based approaches authenticating a device, depending on its software implementation or protocol characteristics, using MAC frames or network traffic [15,16]. On the other hand, RF fingerprinting authenticates or identifies a device using its components impairments (hardware-based) or channel characteristics (channel-based).

Classically, RF fingerprinting techniques are considered for authentication and access control [17], but some authors prefer to consider it as a second authentication factor [18]. According to Guo et al. [19], RF fingerprinting is divided into two steps. The first step, called “RF fingerprint extraction”, consists of extracting significant features from the observed signal. The second step, called “RF fingerprint authentication”, uses the extracted features to perform authentication.

 One on hand, the channel (or location)-based approaches perform authentication using channel characteristics such a received signal strength (RSS) or channel state information (CSI) [4]. On the other hand, hardware (or radiometric)-based authentication methods exploit emitter impairments to perform authentication [20]. Indeed, the analog front end of an emitter is composed of several components, such as digital-to-analog converters (DAC), I/Q mixer, power amplifier (PA), and local oscillator (LO). The variabilities of the manufacturing process create small impairments on components such as I/Q offset, I/Q imbalance, nonlinear distorsions, and carrier frequency offset (CFO) [21]. While an emitter is transmitting, the impairments of its components create unique signature on the transmitted RF signal. Thus, this signature can be exploited to authenticate a device using its components’ impairments.

 According to Soltanieh et al. [17], the hardware-based approaches are usually divided into two groups: (1) transient-based and (2) steady-state/modulation-based. Transient-based approaches used transitory signals to perform authentication [22,23,24]. For example, Aghnaiya et al. use variational mode decomposition (VMD) and higher-order statistics (HOS) to perform authentication of Bluetooth devices using signal transients [24]. On the contrary, modulation-based approaches are based on steady-state signal, i.e., the modulation part [20,25]. For example, Brik et al. use features such as CFO and I/Q offset extracted from steady-state signals to perform authentication of devices [20].

### 2.3. Feature-Learning for RF Fingerprinting

In recent years, feature learning (or representation learning [26]) is becoming the new trend in RF fingerprinting domains. Indeed, these approaches learn significant features directly from signals rather than using expert knowledge features depending on a priori assumptions [5]. From this perspective, DARPA agency launched, in 2017, a program called Radio Frequency Machine Learning Systems (RFMLS), aiming to develop the use of machine learning for radio frequency [27,28]. Particularly, one task of the program was dedicated to the development of feature-learning algorithms for RF fingerprinting. Recently, deep learning architectures ( according to Goodfellow et al., deep learning methods correspond to a subset of representation learning methods [26]) have been widely used for RF fingerprinting feature learning in modulation-based domains. In [21], Riyaz et al. propose a deep learning architecture for RF fingerprinting based on modulation identification works presented in [29]. Since then, several authors have explored the RF fingerprinting method for IoT using feature learning approaches [6,30,31]. Particularly, many works address the complexity problem in IoT context using a lightweight method [32,33,34]. For example, in [32], the authors present an lightweight procedure based on mobile edge computing (MEC) in IoT context. Furthermore, some authors mentioned that another important aspect of RF fingerprinting for IoT is scalability [5,33], i.e., the capacity of the algorithm to be retrained easily. Indeed, the authors mentioned that Siamese network architectures can solve this problematic, especially using one-shot learning.

## 3. Methodology

The RF eigenfingerprint method presented in this paper is a feature-learning approach on steady-state signal based on the eigenfaces works previously introduced. To do so, the preamble is used to learn the features called RF eigenfingerprints corresponding to the emitter signature. A preamble is a known signal, starting every wireless communication and depending on a specific protocol. Generally, preambles are used for temporal synchronization, frequency synchronization, and equalization. In our case, using a preamble allows us to have a common signal for all wireless devices using the same protocol, reducing the sources of non-significant variability, i.e., the transmitted data.

**Figure 2:**
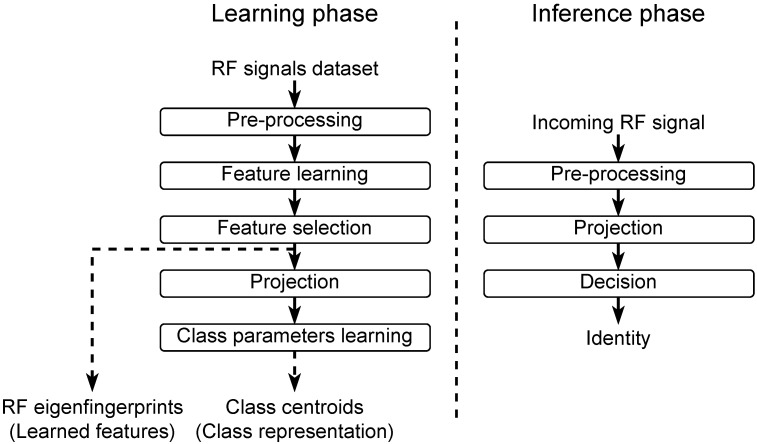
Methodology summary.

In this section, we present different steps necessary for learning or inference. The steps are presented in chronological order, for example, the feature learning (Section 3.2) is before feature selection (Section 3.3). Furthermore, some steps are necessary for learning and inference, such as preprocessing (Section 3.1) and projection (Section 3.4.1). Figure 2 resume the different steps involved during learning phase and inference phase.

### 3.1. Preprocessing

The preprocessing is a step primordial for reducing non-informative variabilities (or factors of variation [26]) present in the signal. Indeed, RF eigenfingerprints are based on singular value decomposition (SVD), a linear algebra tool similar to PCA. As explained by Brunton in [10], SVD and PCA are really sensitive to variability. For example, in eigenfaces, misaligned face datasets (translation, rotation, etc.) require more eigenfaces to learn the face variability than well-aligned face datasets. This feature explosion is due to the fact that PCA (or SVD) is not invariant to scale, translation, and rotation, among others. In the eigenfaces literature, this preprocessing is called data alignment [10]. Thus, performing preprocessing on the received signal is paramount to eliminate non-informative source of variabilities such as signal delay or complex amplitude.

In our case, we consider that the received signal r(t) is the following:

(1) $$r\left(t\right)=As\left(t-\tau \right)e^{j2\pi \Delta ft}+n\left(t\right)$$

where:  

- s(t): The emitted preamble.
- A∈C: The signal complex amplitude.
- τ: The signal delay between emitter and receiver.
- Δf: The frequency offset between emitter and receiver.
- $n\left(t\right)∼\mathrm{CN}\left(0,\sigma^{2}\right)$: An additive white Gaussian complex noise with power $\sigma^{2}$.

**Figure 3:**
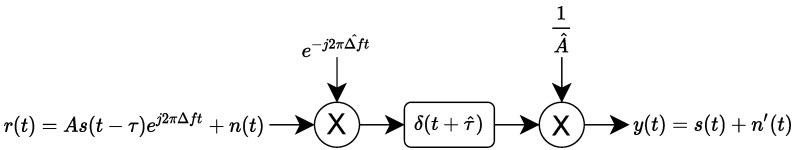
Preprocessing process n°1.

**Figure 4:**
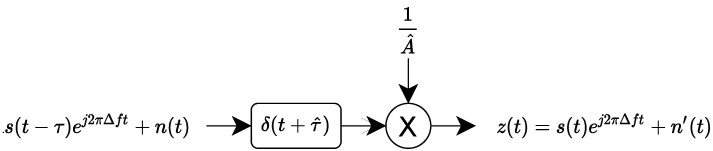
Preprocessing process n°2.

In this paper, we present two preprocessing processes:

- Preprocessing process n°1: This preprocessing process is presented in Figure 3 and consists of correcting the frequency offset, the signal delay, and the complex amplitude.
- Preprocessing process n°2: This preprocessing process is presented in Figure 4 and consists of correcting the signal delay and the complex amplitude. 

The preprocessing process n°1 uses several steps to perform corrections on received signal r(t). The first step of this preprocessing process estimates the frequency offset Δf using the M-th power estimator $r{\left(t\right)}^{M}\approx A^{M}e^{j2M\pi \Delta ft}+n_{1}\left(t\right)$ (with $n_{1}\left(t\right)$ a noise) and Fourier transform (FT): $\hat{\Delta f}=arg\;max_{f}FT\left(s{\left(t\right)}^{M}\right)$. Once the frequency offset is estimated, the preprocessing process compensates frequency offset by multiplying r(t) with a complex exponential $e^{-j2\pi \hat{\Delta f}t}$ resulting in $s_{1}\left(t\right)=As\left(t-\tau \right)+n_{2}\left(t\right)$ (with $n_{2}\left(t\right)$ a noise with the same properties as n(t)). The second step estimates the signal delay τ and the complex amplitude *A* using a cross correlation $R_{s_{1}s}\left(T\right)$ between $s_{1}\left(t\right)$ and s(t). Indeed, the cross-correlation peak allows us to estimate the signal delay $\hat{\tau }=arg\;max_{T}R_{s_{1}s}\left(T\right)$ and the complex amplitude $\hat{A}=max_{T}R_{s_{1}s}\left(T\right)$. Finally, a time shifting and amplitude correction are performed to obtain the centered signal. The final noise $n^{\prime }\left(t\right)$ is an additive white Gaussian complex noise with power $\sigma_{n}^{2}=\frac{\sigma^{2}}{{\hat{A}}^{2}}$. This preprocessing process is similar to some preprocessings present in the RF fingerprinting domain [33].

This preprocessing is really similar to the preprocessing step present before demodulation in wireless communications. Indeed, for data demodulation, frequency synchronization, phase synchronization, and time synchronization are primordial. This common process can be an advantage because it is already required for demodulation and thus adds no additional cost to implement it for RF eigenfingerprints.

The preprocessing process n°2 does not correct frequency offset, considering carrier frequency offset as constant in time [20,31]. Indeed, contrary to preprocessing presented in Figure 3, this preprocessing process estimates the frequency offset Δf but does not perform frequency synchronization. Instead, the estimated frequency offset $\hat{\Delta f}$ is used to obtain a desynchronized preamble $s_{f}\left(t\right)=s\left(t\right)e^{j2\pi \hat{\Delta f}t}$ to estimate signal delay τ and complex amplitude *A*. Indeed, the cross-correlation $R_{rs_{f}}\left(T\right)$ between received signal r(t) and the desynchronized preamble $s_{f}\left(t\right)$ allows us to estimate the signal delay $\hat{\tau }=arg\;max_{T}R_{rs_{f}}\left(T\right)$ and the complex amplitude $\hat{A}=arg\;max_{T}R_{rs_{f}}\left(T\right)$. However, this preprocessing process is different to the demodulation preprocessing step introduced previously and can add additional cost to implement. Identically, the noise $n^{\prime }\left(t\right)$ is an additive white Gaussian noise with power $\sigma_{n}^{2}=\frac{\sigma^{2}}{{\hat{A}}^{2}}$.

In this paper, we propose two preprocessing processes, but other preprocessing processes can be designed for RF eigenfingerprints. For example, equalization can also be part of the preprocessing processes to avoid learned channel-dependent features. Indeed, in the case where the devices are non-static, the channel is another source of variability that requires correction. This preprocessing type has been used by Sankhe et al. [3,35] to compensate the effect of channel for the feature learning RF fingerprinting approach.

### 3.2. Feature Learning

The feature learning approach is performed using SVD and consists of decomposing a matrix *M* as a product of three matrices $M=U\Sigma V^{H}$ [10]. This learning requires a set of preprocessed received signal $x_{i}\in C^{N}$ where *N* is the number of preamble samples. Using these signals, the data matrix $X\in C^{N\times L}$ is obtained and composed of *L* column vectors $x_{i}$:

(2) $$X=\left[\begin{array}{c} x_{1}\cdots x_{L} \end{array}\right]$$

The first step computes the mean vector $\bar{x}\in C^{N}$ composed of the mean of each line ${\bar{x}}_{i}=\sum_{i=1}^{L}x_{ij}$, where $x_{ij}$ is the ith sample of the jth signal $x_{j}$:

(3) $$\bar{x}=\left[\begin{array}{c} {\bar{x}}_{1}\\ \vdots \\ {\bar{x}}_{N} \end{array}\right]$$

Then, the second step computes the centered data matrix $B_{train}\in C^{N\times L}$:

(4) $$B_{train}=X_{train}-\bar{x}\left[1\cdots 1\right]$$

Once the centered data matrix is computed, an SVD is performed, allowing us to obtain the eigenvectors matrix $U=\left[u_{1}\cdots u_{N}\right]\in C^{N\times N}$:

(5) $$B_{train}=U\Sigma V^{H}$$

An additional step can be performed, allowing us to obtain the corresponding eigenvalues $\lambda_{i}$ on the diagonal of matrix $D\in R^{N\times N}$:

(6) $$D=\frac{1}{L-1}\Sigma \Sigma^{T}$$

In this work, we prefer to use SVD instead of PCA, as in eigenfaces works [7,8,11,12]. This implementation choice was made for two principal reasons. The SVD has less computation complexity than PCA and the SVD order eigenvectors, depending on eigenvalues in a descending manner [10].

### 3.3. Features Selection

Classically, features selection in PCA consists of analyzing eigenvalues to find when adding an eigenvector to features subspace is no longer necessary, and is called elbow estimation in the literature [9]. Furthermore, more optimal techniques for eigenvectors selection with SVD are presented in [10]. In this paper, we propose a novel features selection based on the Ljung–Box hypothesis test [36]. This statistical test is classically used in time series to estimate the presence of autocorrelation for lags different from zero. The key idea behind this features selection method is that the first *K* eigenvectors (corresponding to significant features) have non-zero values autocorrelation. In contrast, the last N−K eigenvectors corresponding to white noise have zero values autocorrelation. This type of idea is really similar to signal and noise subspace separation in high-resolution spectral estimation methods such as Pisarenko, MUSIC, and ESPRIT [37]. Algorithm 1 presents the features selection process. It allows us to obtain the matrix $U_{proj}=\left[u_{1}\cdots u_{K}\right]\in C^{N\times K}$, which contains the *K* selected features called the RF eigenfingerprints.  
**Algorithm 1:** Feature eigenvectors selection algorithm
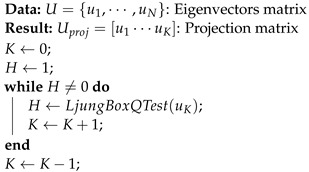


### 3.4. Decision

When RF fingerprinting is used for authentication, it determines if a received signal corresponds to the identity that it claimed (using an ID). Generally, in a IoT network, N devices are allowed to communicate. The outlier detection step determines if the received signal corresponds to one of the known devices. Then, the classification problem consists of identifying the corresponding class.

#### 3.4.1. Projection

Once the projection matrix $U_{proj}$ is obtained, an  preprocessed preamble $x\in C^{N}$ can be projected onto the features subspace to obtain $z\in C^{K}$, as described in Equation (7). This step is called projection but is also referred to as feature extraction [17,19].

(7) $$z=U_{proj}^{H}\left(x-\bar{x}\right)$$

The projected data matrix $Z\in C^{K\times L}$ is obtained similarly:

(8) $$Z=U_{proj}^{H}\left(X-\bar{x}\left[1\cdots 1\right]\right)$$

The projection corresponds to "RF fingerprint extraction" described in Section 2.2.

#### 3.4.2. Statistical Modeling

For the decision step, a statistical modeling of the projected signal *z* is required. To do so, we consider that for a specific class *c*, s(t) is constant, i.e., the impairments are permanent [17]. Furthermore, an additive white Gaussian complex noise $n^{\prime }\left(t\right)∼\mathrm{CN}\left(0,\sigma_{n}^{2}\right)$ projected on an orthonormal basis is also an additive white Gaussian complex noise $n^{{}^{\prime \prime }}\left(t\right)∼\mathrm{CN}\left(0,\sigma_{n}^{2}\right)$ (see Appendix A).

Based on these statements, the statistical modeling of projected signals of a certain class *c* follow a complex multivariate Gaussian distribution [38,39]:

(9) $$f\left(z,\Theta_{c}\right)=\frac{1}{|\Sigma_{c}|\pi^{K}}e^{-{\left(z-\mu_{c}\right)}^{H}\Sigma_{c}^{-1}\left(z-\mu_{c}\right)}$$

where  

- $\mu_{c}$: The mean vector of class *c*.
- $\Sigma_{c}=\sigma_{n}^{2}I$: The variance–covariance matrix of class *c*.
- $\Theta_{c}=\left\{\mu_{c},\Sigma_{c}\right\}$: The distribution parameters of class *c*.  

It can be noted that statistical modeling supposes a homoscedasticity hypothesis between classes, i.e., the variance–covariance matrix is identical for each class. This hypothesis supposes the SNR is similar for all emitters.

Furthermore, the modeling of the *C* known class (with *C* the numbers of class) is a Gaussian mixture model described here:

(10) $$g\left(z,\Theta \right)=\sum_{k=1}^{C}p_{k}f\left(z,\Theta_{k}\right)$$

where  

- $p_{k}=P\left(y=k\right)$: The probability of receiving a signal. In this paper, we consider that the emission probability of a certain emitter is equal to $\frac{1}{C}$. Another possibility is to consider $p_{k}$ in a multinoulli distribution and estimate it using frequentist approach) for class *k* (here, $\frac{1}{C}$).
- $\Theta =\{\Theta_{1},\cdots ,\Theta_{C},p_{1},\cdots ,p_{C}\}$: The mixture parameters.

#### 3.4.3. Class Parameters Learning

The only parameter to learn for each class is the class centroid $\mu_{c}$, using the following estimator:

(11) $${\hat{\mu }}_{c}=\frac{1}{\sum_{i=1}^{L}\delta \left(y_{i},c\right)}\sum_{i=1}^{L}\delta \left(y_{i},c\right)z_{i}$$

where  

- $z_{i}$: The projection of $x_{i}$ in the subspace.
- $y_{i}$: The corresponding class of $x_{i}$.
- δ: The Dirac function.  

Each axis being independent from the others due to projection on an orthonormal basis caused learning of $\mu_{c}$ to not be affected by curse of dimensionality [9].

Furthermore, it is possible to determine the number of examples required to estimate a class centroid knowing the signal-to-noise ratio (and thus the value of $\sigma_{n}^{2}$). Indeed, Appendix B.1 gives the formula to determine the number of samples $L_{c}$ required to estimate a class centroid using a 1−α confidence interval:

(12) $$L_{c}=\frac{t_{1-\alpha }\sigma_{n}^{2}}{2I_{c}}$$

where  

- $t_{1-\alpha }$: The threshold corresponding to $P(X<t_{1-\alpha })=1-\alpha$ (with $X∼\chi^{2}\left(2\right)$).
- $I_{c}$: The confidence interval size.  

The confidence interval size can be defined as a fraction of $d=\lambda_{min}-\sigma_{n}^{2}$, where $\lambda_{min}$ is the eigenvalue corresponding to the last selected eigenvectors (see Appendix B.2). Indeed, the eigenvalue $\lambda_{i}$ represents the variance in projected axis *i*, and $\sigma_{n}^{2}$ represents the intra-class variation. Thus, the term $d=\lambda_{min}-\sigma_{n}^{2}$ represents the inter-class variation, i.e., the variation of class means in the last selected eigenvector. Finally, defining confidence interval size as a fraction of *d* could avoid class centroid superposition on the last axis due to lack of examples during estimation.

#### 3.4.4. Outlier Detection

The first step in the RF fingerprinting decision process determines if a given signal *x* and its projection *z* correspond to a legitimate user. Outlier detection for RF eigenfingerprints is described by Equation (13) [9,18]:

(13) δ(g(z,Θ)<T)

where  

- *T*: The outlier tolerance threshold (the outlier tolerance threshold can be determined using the method presented in [9]).
- δ: The indicator function.

#### 3.4.5. Classification

The second step in the RF fingerprinting decision process identifies class belonging to a signal considered as legitimate. The classification is based on maximum a posteriori:

(14) $$y=argmax_{c}\;P\left(y=c|z\right)$$

In our case, the a priori probability is $P\left(y=c\right)=\frac{1}{C}$, i.e., legitimate emitters have same emitting probability. Thus, the maximum a posteriori rule becomes a maximum likelihood rule. Finally, the homoscedasticity hypothesis ($\Sigma_{c}=\sigma_{n}^{2}I$) classification decision becomes a mean centroid classifier:

(15) $$\begin{array}{cc} y & =arg\;max_{c}\;P\left(z|y=c\right) \end{array}$$

(16) $$\begin{array}{cc} \; & =arg\;max_{c}\;f\left(z,\Theta_{c}\right) \end{array}$$

(17) $$\begin{array}{cc} \; & =arg\;max_{c}\;\frac{1}{{\left(\pi \sigma^{2}\right)}^{K}}e^{\frac{{∥z-{\hat{\mu }}_{c}∥}^{2}}{\sigma^{2}}} \end{array}$$

(18) $$\begin{array}{cc} \; & =arg\;max_{c}\;{∥z-{\hat{\mu }}_{c}∥}^{2} \end{array}$$

Another strategy (this strategy must be preferred if SNR of different emitter are not similar) exists, combining outlier detection and classification steps, and is presented in eigenfaces works [11,12]. It compares the projection *z* independently to each class centroid estimation ${\hat{\mu }}_{c}$ using a specific class threshold $T_{c}$ (see Appendix C):

(19) $$\delta ({∥z-{\hat{\mu }}_{c}∥}^{2}<T_{c})$$

The class threshold can be determined using the following equation:

(20) $$T_{c}=\frac{t_{1-\alpha }\sigma_{c}^{2}}{2}\frac{L_{c}+1}{L_{c}}$$

where  

- $t_{1-\alpha }$: The threshold corresponding to $P(X<t_{1-\alpha })=1-\alpha$ with $X∼\chi^{2}\left(2K\right)$).
- $\sigma_{c}^{2}$: The normalized noise power of class *c*.  

However, this strategy requires a threshold $T_{c}$ per class, thus increasing the memory impact.

#### 3.4.6. Clustering

Clustering analyses are unsupervised learning methods that automatically identify groups in data without labels. Classic methods for clustering are hierarchical clustering, K-means, and DBSCAN [9]. In an RF fingerprinting context, clustering can be used to determine from rejected signals (considered as illegitimate users during outlier detection) the number of illegitimate users and their intentions. In [40], the authors used non-parametric clustering to detect attacks in wireless networks among Sybil and masquerade attack. Furthermore, Robyns et al. [18] used DBSCAN for unsupervised RF fingerprinting in a Lora network.

Clustering methods can be used in IoT context to perform reverse engineering of a network attack, allowing us to determine the number of attackers and their intentions. The clustering method will be guided by the statistical modeling of classes described in Equations (9) and (10). Furthermore, non-parametric methods would be preferred, i.e., the number of attackers is automatically learned by a clustering algorithm. Gaussian mixture model can be used taking into account the specific hypothesis ($\Sigma_{c}=\sigma_{c}^{2}I$) with Bayesian Gaussian mixture process [9,40].

## 4. Experiments

### 4.1. Impairments Simulation

**Figure 5:**
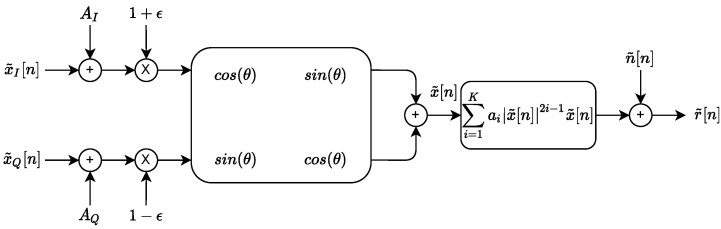
Impairments model.

The first experiment consists of simulating a pool of wireless devices using an RF fingerprinting impairments model presented in Figure 5. This model is inspired by several models present in the literature [25,41,42].

The parameters of the model are the following:  

- IQ offset (*U* is an uniform distribution):
  - $A_{I}∼U\left(-0.01,0.01\right)$: Real part.
  - $A_{Q}∼U\left(-0.01,0.01\right)$: Imaginary part.
- IQ imbalance:
  - ϵ∼U(−0.01,0.01): Gain imbalance.
  - $\theta ∼U(\frac{-\pi }{32},\frac{\pi }{32})$: Phase skew.
- Power amplifier (AM/AM) ($a_{2}$ ans $a_{3}$ are negative and produce amplitude clipping (compression)):
  - K=3.
  - $a_{1}=1$.
  - $a_{2}∼-U\left(\left[-27dB,-33dB\right]\right)$.
  - $a_{3}∼-U\left(\left[-45dB,-55dB\right]\right)$. 

This configuration corresponds to RF eigenfingerprints using preprocessing process n°1 (Figure 3). We simulate 10 different devices with 30 signals for each class. The preamble is a 250-sample QPSK signal with RRC shape filter (the filter is designed with Matlab function rcosdesign (0.5, 4, 5)) with unit power ($P_{s}=1$). The signal-to-noise ratio (SNR) is fixed to 30 dB using additive white Gaussian complex noise ($P_{n}=10^{-3}$).

**Figure 6:**
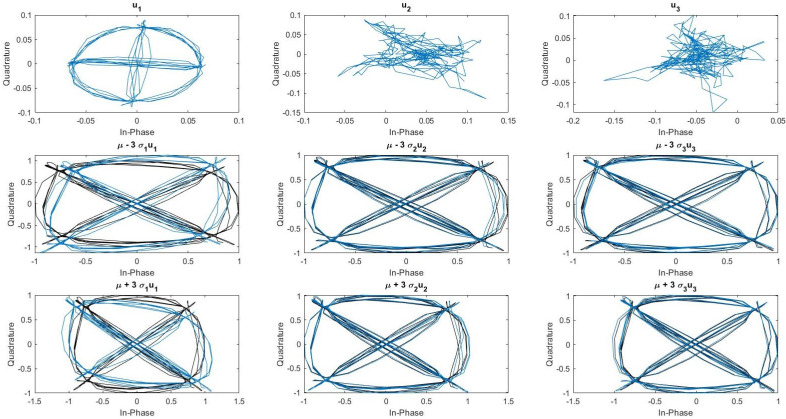
Visualisation of learned features.

The features selection algorithm selects only the first three features. These features are presented in Figure 6. The upper part of the figure corresponds to the learned RF eigenfingerprints $u_{i}$, the middle part corresponds to $\bar{x}-3\sqrt{\lambda_{i}}u_{i}$, and the lower part corresponds to $\bar{x}+3\sqrt{\lambda_{i}}u_{i}$. This technique allows us to evaluate the impact of a specific RF eigenfingerprint $u_{i}$ on the mean signal $\bar{x}$. We can observe that the first RF eigenfingerprint corresponds to I/Q imbalance impairments (ϵ,θ). The second RF eigenfingerprint corresponds to RF power amplifier effect ($a_{2},a_{3}$) and I/Q offset ($A_{I},A_{Q}$). Finally, the last RF eigenfingerprint corresponds also to I/Q offset ($A_{I},A_{Q}$). The learned features have human-level explainability properties compared to other feature learning based on deep learning models. We discussed these explainability properties in Section 5.3.

In addition to feature extraction/learning, explainability and the decision is also interpretable. Indeed, the mean class signal can also be obtained using $s_{c}=\bar{x}+U_{proj}{\hat{\mu }}_{c}$. This signal, composed of mean signal $\bar{x}$, the features subspace $U_{proj}$, and the class centroid estimation ${\hat{\mu }}_{c}$, corresponds to an estimation of the signal sent by the emitter of class *c* (without noise). We also discussed these explainability properties in Section 5.3.

### 4.2. Real-World Performance Evaluation

**Figure 7:**
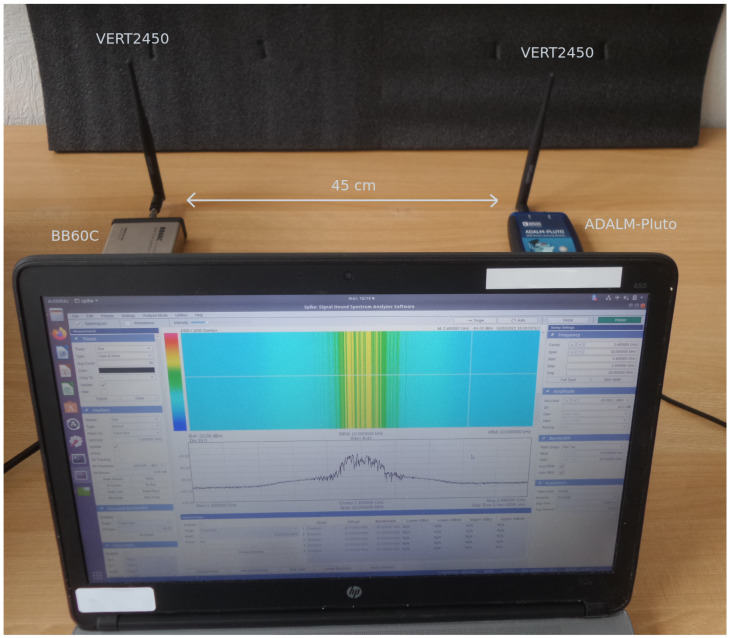
Testbed of the experiment.

In this subsection, we present a real-world experiment showing the classification performances of the RF eigenfingerprints. For this experiment, we used four ADALM-Pluto software-defined radio (SDR) platforms from Analog Devices as emitter, with central frequency $f_{0}$ = 2.45 GHz and sampling frequency $F_{s}$ = 5 Msps (the transmitted signal is a QPSK at 1 MBd with roll-factor β=0.5, thus the signal bandwidth is 1.5 MHz. The authors choose these signal parameters according to WPAN IoT standards such as ZigBee, using VERT2450 antenna (Ettus Research). Concerning the receiver part, we used a BB60C I/Q signal recorder (the software used to record the signals is Spike from Signal Hound) with central frequency $f_{0}$ = 2.45 GHz and frequency sampling $F_{s}$ = 5 Msps using VERT2450 antenna. The signal recorder is located at 45 cm from the emitter. The testbench of the experiment is presented in Figure 7. For each ADALM-Pluto (class), we collect 50 preamble signals (the same as presented in Section 4.1). A preprocessing step is performed consisting of injecting additive white Gaussian complex noise to obtain a desired SNR ($SNR_{d}$) of 30 dB. The procedure used is similar to the noise injection presented in [43]. The only difference is that the power of noise to be injected $n^{\prime }\left(t\right)$ is computed as follows: $P_{n}^{\prime }=\frac{P_{s}}{SNR_{d}}-P_{n}$. Finally, we split the signals into 70/30, leading to 35 training signals and 15 testing signals for each class.

Different classifiers are studied:  

- Classifier 1: RF eigenfingerprints using preprocessing process n°2 (Figure 4).
- Classifier 2: RF eigenfingerprints using preprocessing process n°1 (Figure 3).
- Classifier 3: Naive Bayes classifier composed of RF eigenfingerprints statistical model (Equation (9)) using preprocessing process n°1 and Gaussian distribution for frequency offset.

**Figure 8:**
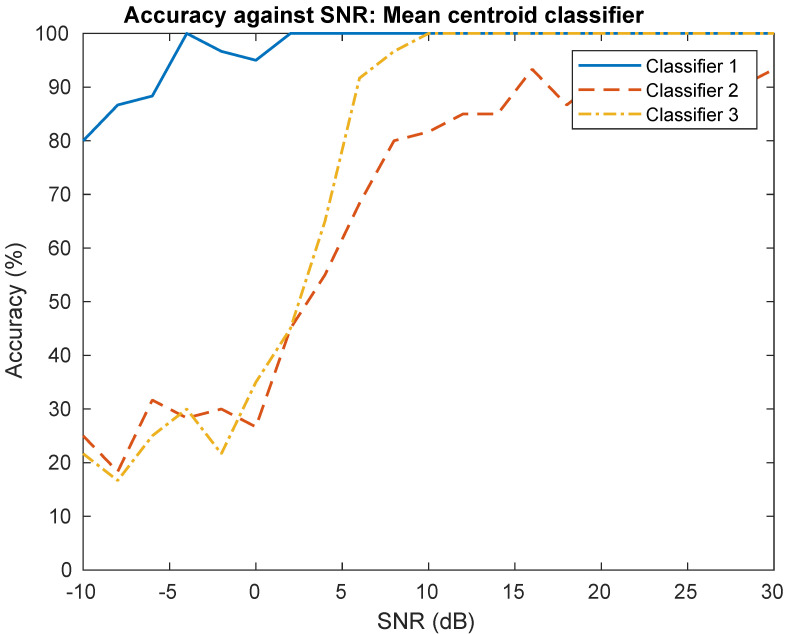
Performances evaluation on real-world signals.

The training is performed with 30 dB SNR on the training set (140 signals). The mean signal is $\bar{x}$, and features subspace $U_{proj}$ and centroid class ${\hat{\mu }}_{c}$ are learned during training. The testing is performed injecting noise on a testing set (60 signals) to obtain a specific SNR from 0 dB to 30 dB. The classification performances are shown in Figure 8. We can observe that the most efficient classifier is classifier 1, with a classification accuracy of nearly 100% from 0 dB to 30 dB. This can be explained because frequency offset is highly significant [20] for classification. Furthermore, it seems more efficient to estimate carrier frequency offset than classifier 3 for low SNR values. Secondly, classifier 3 is also efficient for SNR from 6 dB to 30 dB. Finally, classifier 2 has the lowest classification performances compared to the other classifiers.

The Classifier 1, using preprocessing process n°2, needs more features than other classifiers because it is based on linear combination of features for learned frequency offset concept. Furthermore, the features learned are less interpretable compared to other classifiers presented in Figure 6. Classifier 2 is potentially the better classifier, with a good compromise between explainability, complexity, and performance.

In [21], the authors used CNN on I/Q signals with similar experimental conditions. Particularly, the signals of 128 samples used in this experiment come from five SDR platforms (USRP B210/X310) at 2.45 GHz sampled at 1.92 Msps. For an SNR of 20 dB, their architecture reached an accuracy of 90%. Compared to their results, the proposed classifier 1 and classifier 2 show better performances. In [34], the authors proposed a lightweight CNN architecture for RF fingerprinting of four Zigbee devices. Their architecture showed less noise robustness than classifier 1 but showed better noise robustness than classifier 2 and 3 for low SNR (0–10 dB). It can be noted that other works show better results [3,5] compared to results presented in [21]. However, these approaches are difficult to compare with our results because they considered more emitters than in the experiment presented in Section 4.2.

### 4.3. FPGA Implementation

In this part, we provide an FPGA implementation of projection described by Equation (7) using RF eigenfingeprints learned by classifier 2 and 3 in the previous section (N=250, K=7). This projection is implemented using Vivado High-Level Synthesis (HLS) for Xilinx Zedboard (Zynq-7000) at 80 MHz. Vivado HLS 2019.2 is a high-level synthesis software that produces hardware description level (HDL) code using high-level synthesis language. For our implementation, the high-level language used is C++ and the hardware description level produced is VHDL.

The pseudocode of projection implementation is presented in Algorithm 2. The input $x\in C^{250}$ is a complex array (in reality, a complex array is represented using two real arrays: one array for real part and one array for imaginary part) of 250 samples stored in block RAM (BRAM), and the output $z\in C^{7}$ is a complex array of seven coefficients stored in BRAM. The first step of projection initializes projection vector *z* as a null vector. Secondly, a loop iteratively computes the value of projection vector *z* for each signal sample $x^{\left(i\right)}$. Indeed, each $x^{\left(i\right)}$ is subtracted to obtain $y^{\left(i\right)}=x^{\left(i\right)}-{\bar{x}}^{\left(i\right)}$, the ith sample of centered signal *y*. Then, each coefficient of z is updated using $z^{\left(j\right)}=z^{\left(j\right)}+h_{j,i}^{*}y^{\left(i\right)}$. This iterative process is repeated for each $x^{\left(i\right)}$.

Two optimizations, **opti1** and **opti2**, can be used to accelerate the projection. The optimization **opti1** based on *#pragma HLS unroll* asks the tool to unroll the initialization loop. The optimization **opti2** based on *#pragma HLS pipeline* asks the tool to pipeline the internal projection loop. Further optimizations are possible, but **opti1** and **opti2** give us the best compromise between error reconstruction and hardware resources.   
**Algorithm 2:** Pseudocode of FPGA implementation
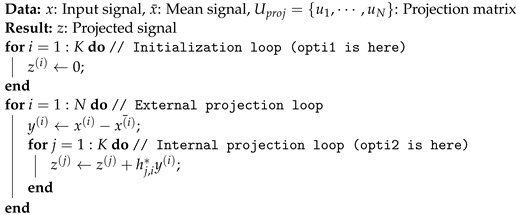


The different implementation results ${\hat{z}}_{proj}$ are compared with Matlab reference $z_{proj}$ result using mean square error (MSE) in percentage $E={∥{\hat{z}}_{proj}-z_{proj}∥}^{2}/{∥{\hat{z}}_{proj}∥}^{2}$. Using ap_fixed<16, 2> (arbitrary precision fixed point, 16 bits), the MSE error in percentage is 0.6294%. Furthermore, different implementations depending on optimizations **opti1** and **opti2** are reported in Table 1.

The most efficient implementation is apfixed162_v3 because it performed projection in 22.03 μs and required less area (BRAM18K, DSP48E, FF, LUT) than apfixed162_v4. The projection implemented is 250 × 7 transform which requires a centering (250 × 1) using mean signal $\bar{x}$ and projection (250 × 7) using projection matrix $U_{proj}$.

On one hand, the signature of an emitter used for this implementation is composed of 224 bits (${\hat{\mu }}_{c}\in C^{7}\to 2\times 7\times 16$ bits). On the other hand, RSA algorithm requires a key size of 1024 bits or bigger. Thus, our implementation reduces, by a factor of at least 4.57, the memory required to store an emitter signature. Furthermore, compared to RSA’s fastest HW/SW implementation on Zynq-7000, the execution time is reduced by a factor of 138 (reference architecture for comparison is apfixed162_v3) [44] and the execution time is reduced by a factor of 77, compared to fastest FPGA implemented presented in [44]. Finally, the area occupied by our implementations is smaller than the majority of implementations presented in [44]. Thus, compared to RSA implementations, our physical-layer authentication implementation requires less memory to store emitter signature, has smaller latency, and requires fewer hardware resources than RSA implementations.

## 5. Interesting Properties in IoT Context

### 5.1. Integration in IoT Networks

Integrating an RF fingerprinting algorithm in an IoT network is a complex challenge. On one hand, an RF fingerprinting algorithm aims to determine if an incoming signal comes from a legitimate emitter at the physical layer. One the other hand, the interactions between the algorithm and the upper layers must also be taken into account. This subsection presents the decision process for RF fingerprinting and its interaction with upper layers.

#### 5.1.1. Three-Steps Decision

**Figure 9:**
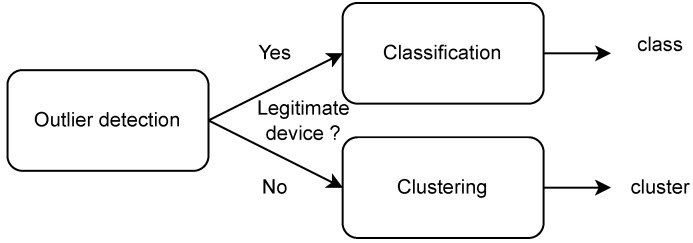
Three-steps decision process.

Three-steps decision (also called 3-steps decision) is a general but important property for RF fingerprinting authentication. As previously explained in Section 3.4, 3-steps decision is important to perform authentication using RF fingerprinting. The first step, called outlier detection, determines if an incoming signal is a legitimate device. On one hand, if the incoming signal is considered to be coming from a legitimate user and is accepted, the second decision step, called classification, identifies among N known devices which one it belongs to. On the other hand, if the incoming signal is considered as an illegitimate device and rejected, clustering can be performed for network attack reverse engineering using all rejected signals to determine the number of attackers and their intentions. The 3-steps detection process is described in Figure 9. It can be noted that a 2-steps decision process can also be implemented for non-cryptographic authentication in an IoT network. This 2-steps decision process, combining outlier detection and classification, allows us to identify if an incoming signal is legitimate and, in this case, to know to which device it corresponds.

#### 5.1.2. Interactions with Upper Layers

Concerning the implementation of the three-steps decision in IoT networks, several aspects should be considered. First of al, Xie et al. have mentioned that during the training phase (or learning phase), the legitimate devices must use the upper-layers authentication mechanisms to prove their identities [45]. Indeed, if an attacker performed an attack during the training phase (referred to as poisoning attack [46]), the performance of RF fingerprinting algorithm during inference phase can be drastically impacted. Moreover, the interactions of the RF fingerprinting algorithm with upper layers during inference phase should also be considered. In [20], the authors propose a server-based implementation of an RF fingerprinting algorithm for WLAN network monitoring. They mentioned that when their system detects illegitimate devices, alerts can be send to network administrators. Alternatively, RF fingerprinting algorithms can be implemented on the IoT devices as second authentication factor [18], i.e., in addition to cryptographic authentication mechanisms.

### 5.2. IoT Properties

The RF fingerprinting algorithm must be designed according to specific properties required to be implemented in IoT networks. Indeed, these networks have specific characteristics that impact the implementation possibilities. This subsection presents these different mandatory properties in an IoT context.

#### 5.2.1. Scalability

Some important characteristics of IoT networks are dynamic changes and enormous scale, among others [1]. Thus, as explained by Shah et al. [2], an important challenge in IoT network is scalability. The scalability for a computer system consists of being able to adapt its size really easily. In IoT context, smart devices must join and quit a network in a simple manner. In our case, this property can be obtained because feature learning (Section 3.2) is independent from decision parameters learning (Section 3.4), contrary to classic deep learning models. Two configurations for feature subspaces learning are possible: (1) learning features on the N known devices dataset [12]; (2) learning features on a general dataset of devices [5]. As we already explained in [5], the scalability for RF fingerprinting can be decomposed into two sub-properties:

- Few-shot learning: This property consists of requiring few data to learn a specific class. Generally, the deep learning model requires at least a thousand data for learning a class. On the contrary, our method requires fewer data per class (35 examples per class).
- Partial retrainability: This property consists of adding or removing a wireless device simply. It is possible because feature learning and feature class parameters learning are independent and classification is not based on a common classifier.

Feature learning approaches generally require a lot of data to be trained. In [21], the authors used 720,000 examples to train their network, i.e., 180,000 examples per class. In [5,33], the authors suggested that Siamese networks could be used to solve this problem of scalability. Indeed, Siamese networks are able to perform one-shot learning, i.e., learn a class representation using a single example. However, these algorithms require a pretraining phase necessary to learn projection space using thousand of training examples. Unlike Siamese networks, our approach requires few examples to learn the projection space and has smaller complexity.

#### 5.2.2. Complexity

Another challenge of IoT networks is device-level energy issues [1]. Indeed, lots of smart devices of sensing layers are low-power technologies and have small computation capacities. Thus, smart IoT devices cannot implement classic cryptography algorithms, such as RSA, among others. Thus, designing small-complexity RF fingerprinting intended for smart devices is primordial. This will allow RF fingerprinting algorithms to be implemented on smart devices as non-cryptographic authentication methods. Similarly to scalability, complexity property can be decomposed into two sub-properties:

- Computation: This property requires that projection and 2-steps decision (projection, classification) are simple to compute. The computation complexity of RF eigenfingerprints are summarized in Table 2 (the definitions of N, K, and C are presented in Section 3).
- Memory: This property requires that features projection and 2-steps decision (projection, classification) have low memory impact. The memory complexity of RF eigenfingerprints are summarized in Table 2.

Feature learning approaches can also have high complexity. For example, in [21], their CNN architecture was composed of more than a hundred thousand parameters. Some authors proposed smaller architecture for feature-learning approaches in RF fingerprinting. Arroyo et al. presented a low complexity trainable architecture with 1757 trainable parameters [33]. For our part, the model trained in Section 4.2 was composed of 2282 complex parameters. However, our method is more scalable than the approach presented in [33] because the number of features, K, does not depend on class number C. Moreover, several authors proposed lightweight CNN architecture for RF fingerprinting in IoT context [33,34]. Although their architectures were smaller than classic CNN architectures, the number of trainable parameters are still around 200,000.

### 5.3. Explainability

Generally, feature learning is performed using deep learning architecture such as convolutive neural networks. However, deep learning networks are considered as black-box models. Even if some efforts are made to understand deep learning in theoretical or practical manners, it is not yet sufficient, thus limiting their usage in critical contexts. Indeed, explainability has close relations with security [46] and is one of the keys for usage of RF fingerprinting in IoT networks. The method we present in this paper has good explainability properties. On one hand, learned features $u_{i}$ called RF eigenfingerprints are interpretable, as observed in Figure 6. On the other hand, class mean signal $s_{c}=\bar{x}+U_{proj}{\hat{\mu }}_{c}$ can also be observed and analyzed using expert knowledge.

Some experiments have been conducted to interpret learned features for modulation recognition tasks, such as those presented in [29,47]. However, to the best of our knowledge, only Kuzdeba et al. have proposed an approach to interpret features learned by their architecture [48] in the RF fingerprinting domain. Besides our learned features explainability, our method has better theoretical background than their method.

## 6. Conclusions

This paper presents the RF eigenfingerprints method based on face recognition works called eigenfaces. Our method consists of learning features using singular value decomposition (SVD), selecting important ones using the Ljung–Box hypothesis test and performing decision based on statistical modeling. We present several experiments showing properties and performances of RF eigenfingerprints. Particularly, we provide simulation using a novel RF fingerprinting impairments model. Using this experiment, the authors present a methodology allowing human-level explainability of learned features. Furthermore, we also provide real-world experiment based on ADALM-Pluto SDR platforms. Our method shows good classification performances, even for low SNR using few examples (dozens of examples per class). Finally, we provide FPGA implementation of feature extraction, called projection, on Zedboard using Vivado HLS. Our FPGA implementation performed feature extraction in about 22 μs with a small hardware area. Our method presents good explainability properties from a theoretical or experimental point of view. It is also a good compromise between high scalability and low complexity, making it well adapted in an IoT context. Future research need to be focused on RF fingerprinting pairing protocols, allowing smart devices to be registered securely on IoT network, avoiding data poisoning attack. Next works could be focused on (1) evaluating the performance of the algorithm for bigger number of emitters, (2) exploring the case of additive non-Gaussian noise, and (3) developing RF fingerprinting pairing protocols for secure registration of smart devices on IoT networks. 

## Figures and Tables

**Table 1:** Implementations comparison.

Version	opti1	opti2	BRAM18K	DSP48E	FF	LUTs	Cycles	Latency
apfixed162_v1	No	No	6	4	224	363	6009	75.11 μs
apfixed162_v2	Yes	No	6	4	223	417	6004	75.05 μs
apfixed162_v3	No	Yes	6	5	266	446	1762	22.03 μs
apfixed162_v4	Yes	Yes	6	5	265	547	1757	21.96 μs

**Table 2:** RF eigenfingerprints complexity.

Type	Computation	Memory
Projection	O(NxK)	O(NxK)
Classification	O(KxC)	O(KxC)

## Data Availability

Not applicable.

## Appendix A. Noise Projection on Orthonormal Basis

In wireless communication, the additive noise is due to thermal noise and generally modeling using an additive white Gaussian complex noise [49]. Indeed, the thermal noise is linked to thermal agitation of charges. Under 100 GHz, the thermal noise has white properties, meaning that the spectral density can be considered as flat. Furthermore, considering the large number of charges, the density probability of thermal noise is considered Gaussian [50].

This appendix presents the main steps of the demonstration, where a white Gaussian complex noise vector $n={\left(\begin{array}{c} n_{1}\cdots n_{N} \end{array}\right)}^{T}$ projected on an orthonormal basis *U* is still a white Gaussian complex noise vector $n_{p}={\left(\begin{array}{c} n^{\left(1\right)}\cdots n^{\left(N\right)} \end{array}\right)}^{T}$ (with $n^{\left(k\right)}=\sum_{i=1}^{N}\alpha_{i}\left(k\right)n_{i}$ and $\alpha_{i}\left(k\right)$ the ith coefficient of axis k of orthonormal basis *U*) validating the statistical modeling presented in Equation (9). The different steps of the demonstration are the following:

1. Demonstrating that $\forall \left(i,j\right),Cov\left(n^{\left(i\right)},n^{(j}\right)=\delta \left(i,j\right)\sigma_{n}^{2}$ allows us to prove that noise projection on different axes are statistically independent.
2. Demonstrating that $\forall i,Cov\left(ℜ\left(n^{(i}\right)\right),ℑ\left(n^{(i}\right))=0$ and $ℜ\left(n^{(i}\right))∼N\left(0,\frac{\sigma_{n}^{2}}{2}\right)$ and $ℑ\left(n^{(i}\right))∼N\left(0,\frac{\sigma_{n}^{2}}{2}\right)$ (using Lindenberg conditions) prove that $n^{\left(i\right)}∼\mathrm{CN}\left(0,\sigma_{n}^{2}\right)$.
3. Demonstrating that $\forall \left(i,j\right),Cov\left(n^{\left(i\right)},n^{(j}\right)=\delta \left(i,j\right)\sigma_{n}^{2}$ and $n^{\left(i\right)}∼\mathrm{CN}\left(0,\sigma_{n}^{2}\right)$ prove the statistical distribution of noise vector projection $n_{p}=U^{H}n\in C^{N}$ of complex Gaussian noise vector $n\in C^{N}$ can be described by (with $\Sigma_{c}=I\sigma_{n}^{2}$):
  (A1) $$f_{n_{p}}\left(n_{p},\Sigma_{c}\right)=\frac{1}{|\Sigma_{c}|\pi^{N}}e^{-n_{p}^{H}\Sigma_{c}^{-1}n_{p}}$$

It can be noted that Tse has demonstrated, in [49], that a white Gaussian noise projected on an orthogonal basis is still a white Gaussian noise on the projected space.

## Appendix B. Class Centroid Sampling

### Appendix B.1. Determining Sample Size

For a specific class, we consider ${\hat{\mu }}_{c}$ as the mean centroid estimation of class *c*. Particularly, we note ${\hat{\mu }}_{c}^{\left(i\right)}$ the ith component of the estimation corresponding of the ith axis. In this appendix, we will define ${\hat{\mu }}_{c}$ as follows:

(A2) $${\hat{\mu }}_{c}=\frac{1}{L_{c}}\sum_{j=1}^{L_{c}}z_{k}$$

where:

- $z_{j}$: The projection of an aligned signal $x_{j}$ belonging to class *c* in the subspace.
- $L_{c}$: The samples number used to estimate the class centroid $\mu_{c}$.

As previously explained, each axis is independent from the others because of the projection in orthonormal basis. Here, we will demonstrate for a specific axis *i* and a specific class *c*, but the demonstration is identical for all axes. We note $z_{j}^{\left(i,c\right)}=\left(\mu^{\left(i,c\right)}+z_{n}\right)∼\mathrm{CN}\left(\mu^{\left(i,c\right)},\sigma_{n}^{2}\right)$ the projection of signal $x_{j}$ belonging to class *c* on the axis *i* and $z_{n}∼\mathrm{CN}\left(0,\sigma_{n}^{2}\right)$. We are interested in estimating the variation of the following estimator:

(A3) $${\hat{\mu }}_{c}^{\left(i\right)}=\frac{1}{L_{c}}\sum_{j}z_{j}^{\left(i,c\right)}$$

where $L_{c}$ is the number of examples used for the estimation.

Thus, ${\hat{\mu }}_{c}^{\left(i\right)}=\left(\mu^{\left(i,c\right)}+{\bar{z}}_{n}\right)∼\mathrm{CN}\left(\mu^{\left(i,c\right)},\frac{\sigma_{n}^{2}}{L_{c}}\right)$ with ${\bar{z}}_{n}∼\mathrm{CN}\left(0,\frac{\sigma_{n}^{2}}{L_{c}}\right)$. We want to estimate the variance of the estimator ${\hat{\mu }}_{c}^{\left(i\right)}$ and determine a confidence interval. This variance, equal to $\frac{\sigma_{n}^{2}}{L_{c}}$, is characterized entirely by variation of ${\bar{z}}_{n}$. The term ${\bar{z}}_{n}={\bar{x}}_{n}+i{\bar{y}}_{n}$ can be decomposed in real part ${\bar{x}}_{n}∼N\left(0,\frac{\sigma_{n}^{2}}{2L_{c}}\right)$ and imaginary part ${\bar{y}}_{n}∼N\left(0,\frac{\sigma_{n}^{2}}{2L_{c}}\right)$ (${\bar{x}}_{n}$ and ${\bar{y}}_{n}$ are independent).

In this demonstration, we want to find $I_{c}$ such as $P(|{\bar{z}}_{n}{|}^{2}<I_{c})=1-\alpha$. The term $|{\bar{z}}_{n}{|}^{2}={\bar{x}}_{n}^{2}+{\bar{y}}_{n}^{2}$ can be decomposed as a sum of the squared value of its real and imaginary part. Using this, we can determine $I_{c}$ as follows:

(A4) $$P({\left(\frac{{\bar{x}}_{n}}{\sigma_{n}/\sqrt{2L_{c}}}\right)}^{2}+{\left(\frac{{\bar{y}}_{n}}{\sigma_{n}/\sqrt{2L_{c}}}\right)}^{2}\leq t_{1-\alpha })=1-\alpha$$

where $t_{1-\alpha }$ is the threshold such as $P(X<t_{1-\alpha })=1-\alpha$ with $X∼\chi^{2}\left(2\right)$.

The random variables $\frac{{\bar{x}}_{n}}{\sigma_{n}/\sqrt{2L_{c}}}∼N\left(0,1\right)$ and $\frac{{\bar{y}}_{n}}{\sigma_{n}/\sqrt{2L_{c}}}∼N\left(0,1\right)$ follow a standard normal distribution. Thus, the random variable ${\left(\frac{{\bar{x}}_{n}}{\sigma_{n}/\sqrt{2L_{c}}}\right)}^{2}+{\left(\frac{{\bar{y}}_{n}}{\sigma_{n}/\sqrt{2L_{c}}}\right)}^{2}∼\chi^{2}\left(2\right)$. Therefore, the threshold $I_{c}$ can be found as follows:

(A5) $$\begin{array}{cc} P({\left(\frac{{\bar{x}}_{n}}{\sigma_{n}/\sqrt{2L_{c}}}\right)}^{2}+{\left(\frac{{\bar{y}}_{n}}{\sigma_{n}/\sqrt{2L_{c}}}\right)}^{2}<t_{1-\alpha }) & =P({\bar{x}}_{n}^{2}+{\bar{y}}_{n}^{2}\leq \frac{t_{1-\alpha }\sigma_{n}^{2}}{2L_{c}}) \end{array}$$

(A6) $$\begin{array}{cc} \; & =1-\alpha \end{array}$$

Finally, with a confidence interval size $I_{c}=\frac{t_{1-\alpha }\sigma_{n}^{2}}{2L_{c}}$, the minimum number of examples giving a specific interval size $I_{c}$ and a specific probability 1−α is

(A7) $$L_{c}=\frac{t_{1-\alpha }\sigma_{n}^{2}}{2I_{c}}$$

### Appendix B.2. Determining Confidence Interval Size

As previously explained, the confidence interval size can be determined as a fraction of $d=\lambda_{min}-\sigma_{n}^{2}$ to limit estimation variation in the last axis. As previously shown, we will focuse on a single axis *i*, but the demonstration is identical for the others axes.

We will consider $z^{\left(i\right)}$ a random variable following a complex Gaussian mixture GMM(Θ) where $\Theta =\{p_{1},\cdots ,p_{K},\mu_{1}^{\left(i\right)},\cdots ,\mu_{K}^{\left(i\right)},\sigma_{n}^{2}\}$. Furthermore, $z^{\left(i\right)}=x^{\left(i\right)}+iy^{\left(i\right)}$ can be decomposed into a real part $x^{\left(i\right)}$ and an imaginary part $y^{\left(i\right)}$, both independent and following a real Gaussian mixture. We want to evaluate the variance of $z^{\left(i\right)}$:

(A8) $$\begin{array}{cc} Var(z^{\left(i\right)}) & =Var\left(x^{\left(i\right)}\right)+Var\left(y^{\left(i\right)}\right) \end{array}$$

(A9) $$\begin{array}{cc} \; & =\lambda_{i} \end{array}$$

For this part of the demonstration, we will compute the variance of $x^{\left(i\right)}$, but the demonstration is identical for $y^{\left(i\right)}$. The probability distribution of $x^{\left(i\right)}$ is equal to $g\left(x,\Theta^{I}\right)=\sum_{k}p_{k}f\left(x,\Theta_{k}^{I}\right)$ where $\Theta_{k}^{I}=\left\{\mu_{1}^{I},\cdots ,\mu_{K}^{I},\frac{\sigma_{n}^{2}}{2}\right\}$ with $\mu_{1}^{I}=ℜ\left(\mu_{1}\right)$ Thus, using the König–Huygens theorem:

(A10) $$Var\left(x^{\left(i\right)}\right)=E\left({\left(x^{\left(i\right)}\right)}^{2}\right)-E{\left(x^{\left(i\right)}\right)}^{2}$$

Calculation of $E(x^{\left(i\right)})$:

(A11) $$E\left(x^{\left(i\right)}\right)=\sum_{k}p_{k}\mu_{k}^{I}$$

For the calculation of $E({\left(x^{\left(i\right)}\right)}^{2})$, we will first need to obtain $E({\left(x_{k}^{\left(i\right)}\right)}^{2})$ where $x_{k}^{\left(i\right)}$ is a random variable depending on class *k*. Furthermore, we will decompose $x_{k}^{\left(i\right)}=\mu_{k}^{I}+\frac{\sigma_{n}}{\sqrt{2}}x_{u}∼N\left(\mu_{k}^{I},\frac{\sigma_{n}^{2}}{2}\right)$ with $x_{u}∼N\left(0,1\right)$. Thus, $E\left({\left(x_{k}^{\left(i\right)}\right)}^{2}\right)={\left(\mu_{k}^{I}\right)}^{2}+\frac{\sigma_{n}^{2}}{2}$ because $E(x_{u}^{2})=1$.

Using $E({\left(x_{k}^{\left(i\right)}\right)}^{2})$, we are able to obtain $E({\left(x^{\left(i\right)}\right)}^{2})$:

(A12) $$E\left({\left(x^{\left(i\right)}\right)}^{2}\right)=\sum_{k}p_{k}{\left(\mu_{k}^{I}\right)}^{2}+\frac{\sigma_{n}^{2}}{2}$$

As $Var\left(x^{\left(i\right)}\right)=E\left({\left(x^{\left(i\right)}\right)}^{2}\right)-E{\left(x^{\left(i\right)}\right)}^{2}$, we can obtain the expression of the variance:

(A13) $$\begin{array}{cc} Var(x^{\left(i\right)}) & =\sum_{k}p_{k}{\left(\mu_{k}^{I}\right)}^{2}+\frac{\sigma_{n}^{2}}{2}-{\left(\sum_{k}p_{k}\mu_{k}^{I}\right)}^{2} \end{array}$$

(A14) $$\begin{array}{cc} \; & =\sum_{k}p_{k}{\left(\mu_{k}^{I}-\left(\sum_{j}p_{j}\mu_{j}^{I}\right)\right)}^{2}+\frac{\sigma_{n}^{2}}{2} \end{array}$$

As the demonstration for $Var(y^{\left(i\right)})$ is identical, we can obtain $Var(y^{\left(i\right)})$ as follows:

(A15) $$Var\left(y^{\left(i\right)}\right)=\sum_{k}p_{k}{\left(\mu_{k}^{Q}-\left(\sum_{j}p_{j}\mu_{i}^{Q}\right)\right)}^{2}+\frac{\sigma_{n}^{2}}{2}$$

where $\mu_{k}^{Q}=ℑ\left(\mu_{k}\right)$.

Finally, $Var(z^{\left(i\right)})$ is equal to

(A16) $$\begin{array}{cc} Var(z^{\left(i\right)}) & =\sum_{k}p_{k}\left({\left(\mu_{k}^{I}-\left(\sum_{j}p_{j}\mu_{i}^{I}\right)\right)}^{2}+{\left(\mu_{k}^{Q}-\left(\sum_{j}p_{j}\mu_{i}^{Q}\right)\right)}^{2}\right)+\sigma_{n}^{2} \end{array}$$

(A17) $$\begin{array}{cc} \; & =d+\sigma_{n}^{2} \end{array}$$

The term $d=\sum_{k}p_{k}\left({\left(\mu_{k}^{I}-\left(\sum_{j}p_{j}\mu_{i}^{I}\right)\right)}^{2}+{\left(\mu_{k}^{Q}-\left(\sum_{j}p_{j}\mu_{i}^{Q}\right)\right)}^{2}\right)$ corresponds to the inter-class variation, i.e., the variation of means of each class in the projected axis *i*. Thus, $d=\lambda_{i}-\sigma_{n}^{2}$ represents the inter-class variation and it can be used to determine the value of confidence interval size $I_{c}$.

## Appendix C. Class Threshold Computing

This appendix focuses on explaining the procedure to find the class threshold $T_{c}$. This threshold is computed to obtain $P({∥z-{\hat{\mu }}_{c}∥}^{2}<T_{c})=1-\alpha$ with z being a projected signal from class *c*.

The vector $z=\mu_{c}+z_{n}$ with $z_{n}=\left(\begin{array}{c} z_{n}^{\left(1\right)}\\ \cdots \\ z_{n}^{(}K) \end{array}\right)\in C^{K}$ and $\forall i,\;z_{n}^{\left(i\right)}∼\mathrm{CN}\left(0,\sigma_{c}^{2}\right)$ and $\forall \left(i,j\right),\;Cov\left(z_{n}^{\left(i\right)},z_{n}^{\left(j\right)}\right)=\delta \left(i,j\right)\sigma_{c}^{2}$. Furthermore, the vector ${\hat{\mu }}_{c}=\mu_{c}+\bar{z}$ with $\bar{z}=\left(\begin{array}{c} {\bar{z}}^{\left(1\right)}\\ \cdots \\ {\bar{z}}^{(}K) \end{array}\right)\in C^{K}$ and $\forall i,\;{\bar{z}}^{\left(i\right)}∼\mathrm{CN}\left(0,\frac{\sigma_{c}^{2}}{L_{c}}\right)$ and $\forall \left(i,j\right),\;Cov\left({\bar{z}}^{\left(i\right)},{\bar{z}}^{\left(j\right)}\right)=\delta \left(i,j\right)\frac{\sigma_{c}^{2}}{L_{c}}$. Thus, the distance between *z* and ${\hat{\mu }}_{c}$ is the following:

(A18) $$\begin{array}{cc} {∥z-{\hat{\mu }}_{c}∥}^{2} & =\sum_{i=1}^{K}{\left|z_{n}^{\left(i\right)}-{\bar{z}}^{\left(i\right)}\right|}^{2} \end{array}$$

(A19) $$\begin{array}{cc} \; & =\sum_{i=1}^{K}ℜ{\left(z_{n}^{\left(i\right)}-{\bar{z}}^{\left(i\right)}\right)}^{2}+ℑ{\left(z_{n}^{\left(i\right)}-{\bar{z}}^{\left(i\right)}\right)}^{2} \end{array}$$

As previously explained, $z_{n}^{\left(i\right)}∼\mathrm{CN}\left(0,\sigma_{c}^{2}\right)\Leftrightarrow ℜ\left(z_{n}^{\left(i\right)}\right)∼N\left(0,\frac{\sigma_{c}^{2}}{2}\right)\;and\;ℑ\left(z_{n}^{\left(i\right)}\right)∼N\left(0,\frac{\sigma_{c}^{2}}{2}\right)\;and\;Cov\left(ℜ\left(z_{n}^{\left(i\right)}\right),ℑ\left(z_{n}^{\left(i\right)}\right)\right)=0$ (identically for ${\bar{z}}^{\left(i\right)}$). Thus, as $z_{n}^{\left(i\right)}$ and ${\bar{z}}^{\left(i\right)}$ are independent, the random variable $z_{c}^{\left(i\right)}=\left(z_{n}^{\left(i\right)}-{\bar{z}}^{\left(i\right)}\right)∼\mathrm{CN}\left(0,\sigma_{c}^{2}\frac{L_{c}+1}{L_{c}}\right)$. Furthermore, as a squared sum of K independent standard normal random variables, $s_{i}$ follow a chi-squared distribution $\chi^{2}\left(K\right)$, thus

(A20) $$\frac{{∥z-{\hat{\mu }}_{c}∥}^{2}}{\frac{L_{c}+1}{L_{c}}\sigma_{c}^{2}/2}=\sum_{i=1}^{K}\;\frac{ℜ{\left(z_{c}^{\left(i\right)}\right)}^{2}}{\frac{L_{c}+1}{L_{c}}\sigma_{c}^{2}/2}+\frac{ℑ{\left(z_{c}^{\left(i\right)}\right)}^{2}}{\frac{L_{c}+1}{L_{c}}\sigma_{c}^{2}/2}∼\chi^{2}\left(2K\right)$$

Thus, $P(\frac{{∥z-{\hat{\mu }}_{c}∥}^{2}}{\frac{L_{c}+1}{L_{c}}\sigma_{c}^{2}/2}<t_{1-\alpha })=1-\alpha$, with $t_{1-\alpha }$ the threshold, such as $P(X<t_{1-\alpha })=1-\alpha$.

The probability that ${∥z-{\hat{\mu }}_{c}∥}^{2}$ is below a certain threshold with probability 1−α is

(A21) $$P({∥z-\mu_{c}∥}^{2}<\frac{t_{1-\alpha }\sigma_{c}^{2}}{2}\frac{L_{c}+1}{L_{c}})=1-\alpha$$

Finally, the threshold value is obtained as follows:

(A22) $$T_{c}=\frac{t_{1-\alpha }\sigma_{c}^{2}}{2}\frac{L_{c}+1}{L_{c}}$$
